# Impact of cirrhosis aetiology on incidence and prognosis of hepatocellular carcinoma diagnosed during surveillance

**DOI:** 10.1016/j.jhepr.2021.100285

**Published:** 2021-03-26

**Authors:** Nathalie Ganne-Carrié, Pierre Nahon, Cendrine Chaffaut, Gisèle N’Kontchou, Richard Layese, Etienne Audureau, Sylvie Chevret, CIRRAL group, Nathalie Ganne-Carrié, Cendrine Chaffaut, Isabelle Archambeaud, Louis d’Alteroche, Frédéric Oberti, Dominique Roulot, Christophe Moreno, Alexandre Louvet, Thông Dao, Romain Moirand, Odile Goria, Eric Nguyen-Khac, Nicolas Carbonell, Jean-Charles Duclos-Vallée, Stanislas Pol, Victor de Ledinghen, Violaine Ozenne, Jean Henrion, Jean-Marie Péron, Albert Tran, Gabriel Perlemuter, Xavier Amiot, Jean-Pierre Zarski, Sylvie Chevret, ANRS CO12 CirVir group, Pierre Nahon, Tarik Asselah, Dominique Guyader, Stanislas Pol, Hélène Fontaine, Georges-Philippe Pageaux, Victor De Lédinghen, Denis Ouzan, Fabien Zoulim, Dominique Roulot, Albert Tran, Jean-Pierre Bronowicki, Thomas Decaens, Ghassan Riachi, Paul Calès, Jean-Marie Péron, Laurent Alric, Marc Bourlière, Philippe Mathurin, Sebastien Dharancy, Jean-Frédéric Blanc, Armand Abergel, Olivier Chazouillères, Ariane Mallat, Jean-Didier Grangé, Pierre Attali, Louis d’Alteroche, Claire Wartelle, Thông Dao, Dominique Thabut, Christophe Pilette, Christine Silvain, Christos Christidis, Eric Nguyen-Khac, Brigitte Bernard-Chabert, Sophie Hillaire, Vincent Di Martino

**Affiliations:** 6 AP-HP, Hôpital Avicenne, Service d’Hépatologie, Bobigny, Université Sorbonne Paris Nord, Bobigny et INSERM U1138, Université de Paris, France; 7 SBIM, APHP, Hôpital Saint-Louis, Paris, Inserm, UMR-1153, ECSTRA Team, Paris, France; 8 Liver, CHU, Nantes, France; 9 Liver Unit, University Hospital, Tours, France; 10 Liver Unit, University Hospital, Angers, France; 11 AP-HP, Hôpital Avicenne, Service de Médecine Interne, Bobigny, Université Sorbonne Paris Nord, Bobigny, France; 12 Liver unit, CUB Hôpital Erasme, Université Libre de Bruxelles, Belgium; 13 Liver Unit, University Hospital, Lille, France; 14 Liver Unit, University Hospital, Caen, France; 15 Liver Unit, University Hospital, Rennes, France; 16 Liver Unit, University Hospital, Rouen, France; 17 Liver Unit, University Hospital, Amiens, France; 18 Liver Unit, APHP, CHU Saint-Antoine, Paris, France; 19 Liver Unit, APHP, CHU Paul Brousse, Villejuif, France; 20 Université Paris Descartes; APHP, Liver Unit, Hôpital Cochin, France; 21 INSERM U1223, Institut Pasteur, Paris, France; 22 Hepatology Unit, University Hospital, CHU Bordeaux, France; 23 Liver Unit, APHP, CHU Lariboisière, Paris, France; 24 Liver Unit, University Hospital, Haine Saint-Paul, Belgium; 25 Liver Unit, Universitary Hospital Purpan, University Paul Sabatier III, Toulouse, France; 26 Institut National de la Santé et de la Recherche Médicale (INSERM), U1065, Team 8, “Hepatic Complications in Obesity”, Nice, F-06204, Cedex 3, France; 27 University Hospital of Nice, Digestive Centre, Nice, F-06202, Cedex 3, France; 28 Liver Unit, University Hospital, Béclère, APHP, Clamart, France; 29 Liver Unit, APHP, CHU Tenon, Paris, France; 30 Clinique d’hépato-gastroentérologie pôle Digidune CHU de Grenoble, France; 31 AP-HP, Hôpital Avicenne, Service d’Hépatologie, Bobigny, Université Sorbonne Paris Nord, Bobigny et INSERM U1138, Université de Paris, France; 32 AP-HP, Hôpital Beaujon, Service d’Hépatologie, and University Paris Diderot, Sorbonne Paris Cité, CRI, UMR 1149, France; 33 CHU Pontchaillou, Service d’Hépatologie, Rennes, France; 34 AP-HP, Hôpital Cochin, Département d’Hépatologie et INSERM UMS20 et U1223, Institut Pasteur, Université Paris Descartes, Paris, France; 35 Hôpital Saint Eloi, Service d’Hépatologie, Montpellier, France; 36 Hôpital Haut-Lévêque, Service d’Hépatologie, Bordeaux; 37 Institut Arnaud Tzanck, Service d’Hépatologie, St Laurent du Var, France; 38 Hôpital Hôtel Dieu, Service d’Hépatologie, Lyon, France; 39 AP-HP, Hôpital Avicenne, Service de Medeine Interne, Bobigny, France; 40 CHU de Nice, Service d’Hépatologie, et INSERM U1065, Université de Nice-Sophia-Antipolis, Nice, France; 41 Hôpital Brabois, Service d’Hépatologie, Vandoeuvre-les-Nancy, France; 42 Hôpital Michallon, Service d’Hépatologie, Grenoble, France; 43 Hôpital Charles-Nicolle, Service d’Hépatologie, Rouen, France; 44 CHU d’Angers, Service d’Hépatologie, Angers, France; 45 Hôpital Purpan, Service d’Hépatologie, Toulouse, France; 46 CHU Toulouse, Service de Médecine Interne-Pôle Digestif UMR 152, Toulouse, France; 47 Hôpital Saint Joseph, Service d’Hépatologie, Marseille, France; 48 Hôpital Claude Huriez, Service d’Hépatologie, Lille, France; 49 Hôpital St André, Service d’Hépatologie, Bordeaux, France; 50 Hôpital Hôtel Dieu, Service d’Hépatologie, Clermont-Ferrand, France; 51 AP-HP, Hôpital Saint-Antoine, Service d’Hépatologie, Paris, France; 52 AP-HP, Hôpital Henri Mondor, Service d’Hépatologie, Créteil, France; 53 AP-HP, Hôpital Tenon, Service d’Hépatologie, Paris, France; 54 AP-HP, Hôpital Paul Brousse, Service d’Hépatologie, Villejuif, France; 55 Hôpital Trousseau, Unité d’Hépatologie, CHRU de Tours, France; 56 Hôpital d’Aix-En-Provence, Service d’Hépatologie, Aix-En-Provence, France; 57 Hôpital de la Côte de Nacre, Service d’Hépatologie, Caen, France; 58 AP-HP, Groupe Hospitalier de La Pitié-Salpêtrière, Service d’Hépatologie, Paris, France; 59 CHU Le Mans, Service d’Hépatologie, Le Mans, France; 60 CHU de Poitiers, Service d’Hépatologie, Poitiers, France; 61 Institut Mutualiste Montsouris, Service d’Hépatologie, Paris, France; 62 Hôpital Amiens Nord, Service d’Hépatologie, Amiens, France; 63 Hôpital Robert Debré, Service d’Hépatologie, Reims, France; 64 Hôpital Foch, Service d’Hépatologie, Suresnes, France; 65 Hôpital Jean Minjoz, Service d’Hépatologie, Besançon, France; 1 AP-HP, Hôpitaux Universitaires Paris Seine Saint-Denis, APHP, Liver Unit, Bobigny, France; 2 Université Sorbonne Paris Nord, F-93000 Bobigny, France; 3 Inserm, UMR–1138 «Functional Genomics of solid tumors», Centre de recherche des Cordeliers, Université de Paris, France; 4 SBIM, APHP, Hôpital Saint-Louis, Paris, Inserm, UMR-1153, ECSTRA Team, Paris, France; 5 Santé publique, APHP, hôpital Henri Mondor; Clinical Epidemiology and Ageing EA7376 UPEC, Créteil, France

**Keywords:** alcoholic liver disease, cirrhosis, primary liver cancer, competing risk analysis, ALC, alcohol-related, HCC, hepatocellular carcinoma, HR, hazard ratio, MIX, alcohol and virus-related, US, abdominal ultrasound, VIR, virus-related

## Abstract

**Background & Aims:**

In this study we aimed to analyse the impact of the aetiology of cirrhosis on the incidence, characteristics and prognosis of hepatocellular carcinoma (HCC) diagnosed during a surveillance program.

**Methods:**

Individual data from a randomized trial and 2 prospective cohorts of patients with compensated histologically proven cirrhosis recruited between 2000 and 2016 were pooled. The influence of cirrhosis aetiology on survival after HCC detection was assessed using multivariable regression models.

**Results:**

Among 3,533 patients (1,926 virus [VIR], 1,167 alcohol [ALC], 440 combined [MIX]), 431 were diagnosed with HCC after a median follow-up of 57.1 months. The 5-year HCC incidence was lowest in ALC (VIR 12.6%, ALC 9.1%, MIX 14.3%, *p* = 0.04). At the time of diagnosis, tumour burden and Child-Pugh score were comparable across aetiology groups, but early BCLC stages (0/A) were significantly less frequent in ALC (VIR 80%, ALC 37%, MIX 72%) as a result of worse ECOG performance status. However, similar access to first-line curative HCC treatment was reported across aetiology groups (*p* = 0.68). Median survival after HCC diagnosis was significantly reduced in ALC (VIR 39, ALC 21, MIX 34 months, *p* = 0.02). However, when adjusting for tumour size, ECOG and Child-Pugh score, the aetiology of the underlying cirrhosis no longer had a significant impact.

**Conclusion:**

Compared to patients with virus-related cirrhosis, patients with alcohol-related compensated cirrhosis enrolled in a surveillance program have: i) the lowest 5-year HCC incidence; ii) worse overall prognosis, mostly driven by a poor general condition, despite similar access to first-line curative treatment.

**Lay summary:**

It has been suggested that early detection of hepatocellular carcinoma (HCC) may be futile in patients with alcohol-related cirrhosis. By comparing outcomes in more than 3,000 patients with compensated cirrhosis included in surveillance programs, this study suggests that HCC surveillance enables early diagnosis in most patients with alcohol-related cirrhosis despite a higher competing risk of death in these patients. We also report similar access to first-line curative HCC treatment in these patients compared to those with viral cirrhosis, despite higher rates of comorbidities and impaired liver function. Following HCC detection, the later parameters were major drivers of death irrespective of the cause of cirrhosis.

**Registration:**

CHC2000 (NCT00190385) and CIRRAL (NCT01213927) cohorts were registered at ClinicalTrials.gov and the full protocols are available at the following links (https://clinicaltrials.gov/ct2/show/NCT00190385) and https://clinicaltrials.gov/ct2/show/NCT01213927, respectively). The full CirVir protocol is available via the ANRS Web site (http://anrs.fr).

## Highlights

• Alcohol-related cirrhosis linked to the lowest incidence of HCC, the lowest overall survival and the highest incidence of decompensation.
• Alcohol-related cirrhosis linked to fewer cases of early stage HCC, although tumour burden and Child-Pugh score were comparable across groups.
• Patients with alcohol-related cirrhosis had worse survival after HCC diagnosis than those with virus-related cirrhosis.
• The aetiology of cirrhosis had no impact on survival after HCC diagnosis following adjustment for other potential prognostic factors.

## Graphical abstract

**Figure:**
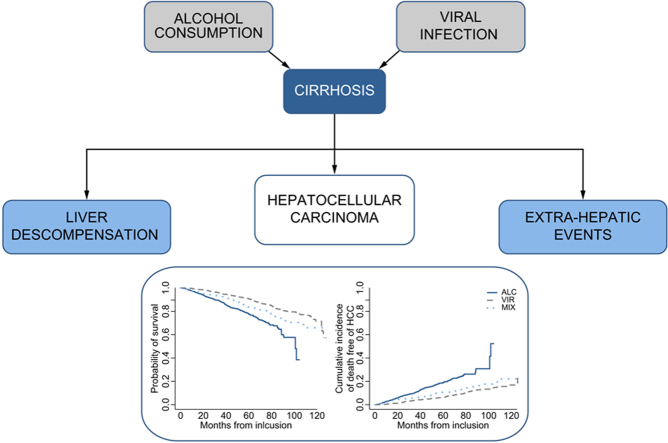

## Introduction

The incidence of hepatocellular carcinoma (HCC) is high in western countries and continues to increase, especially in France, which had over 10,000 cases in 2017.^1^

The contribution of alcohol to global incident cases of liver cancer varies markedly between regions, from 6% in the Middle East, where the leading causes of HCC are HBV and HCV, up to 60% in Eastern Europe, where viral hepatitis is only a small contributor to HCC.^2^ In France, alcohol accounts for at least 37% of HCC cases.

The impact of the underlying liver disease on the characteristics of HCC has been assessed in 2 transversal studies^3^^,^^4^ demonstrating that an alcohol-related aetiology is associated with an adverse prognosis in patients diagnosed with HCC, owing to delays in detecting the cancer, which is frequently diagnosed outside of surveillance programmes, and the fact that the cancer tends to develop in a setting of more advanced chronic liver disease compared to HCV-associated cases. Nevertheless, these studies recruited patients at the time of HCC diagnosis and probably suffered from selection biases since pre-HCC longitudinal follow-up was not analysed.

International guidelines recommend HCC surveillance in all patients with cirrhosis,^5^ including those with alcohol-related liver disease.^6^ However, conflicting data on the incidence of HCC in patients with alcohol-related cirrhosis have triggered controversy regarding the benefits of periodic screening for HCC in this aetiological subgroup.[7], [8], [9], [10] As a whole, regardless of the aetiology of cirrhosis, HCC screening is associated with an earlier stage at diagnosis and an increase in both eligibility for curative treatment and survival.^11^ However, to date, the diagnostic performance of routine screening for HCC according to the aetiology of the underlying cirrhosis has not been evaluated longitudinally.

This prompted us to assess the role of the aetiology of the underlying chronic liver disease on the course of compensated biopsy-proven cirrhosis in terms of baseline presentation and outcome, with a particular focus on HCC detection and subsequent survival.

## Patients and methods

This study used individual data from 1 randomized trial dedicated to HCC surveillance and 2 prospective cohorts of adults with biopsy-proven compensated cirrhosis without any baseline detectable hepatic complications: the CHC2000 trial,^12^ ANRS CO12 ‘‘virus-related cirrhosis” (CirVir) cohort,^13^ and CIRRAL “alcoholic cirrhosis” cohort.^9^ Each study was conducted in accordance with the ethical guidelines of the 1975 Declaration of Helsinki and French law for biomedical research and was approved by the institutional ethics committee (CCPPRB, Aulnay-sous-Bois, France). All patients provided written informed consent to participate.

A standardized follow-up with periodical liver ultrasonography (US) was initiated at the time of liver biopsy showing cirrhosis and prospectively monitored from inclusion in 1 of these 3 studies. In the case of detected focal liver lesions, a recalled diagnostic procedure using contrast-enhanced imaging and/or guided biopsy was performed according to the American Association for the Study of Liver Diseases guidelines.^14^^,^^15^ A diagnosis of HCC was established by either histological examination performed by an experienced pathologist or based on probabilistic non-invasive criteria. HCC treatment was determined using a multidisciplinary approach according to the European Association for the Study of the Liver.^5^

In addition to HCC occurrence, which was the primary endpoint of all 3 cohorts, all events that occurred during follow-up (*i.e.* death, liver decompensation, bacterial infections, extrahepatic malignancies and cardiovascular diseases) were recorded using information obtained from the medical records of the patients held by each centre in the Cirvir and CIRRAL cohorts. All treatments, including antiviral therapies, were recorded at inclusion, and any modifications during follow-up were notified, particularly in the case of severe adverse events.

### CHC 2000

CHC 2000 is a randomized trial conducted in 43 tertiary liver centres in France and Belgium aimed to compare 2 US periodicities for the detection of small HCC ≤30 mm eligible for curative treatment.^12^ The trial, whose promoter was the Assistance Publique-Hôpitaux de Paris (APHP), was funded by the French Ministry of Health (PHRC 1998 and 2003) and the French Ligue de Recherche contre le Cancer (*ClinicalTrials.gov,* NCT00190385). Specific additional inclusion criteria were as follows: i) cause of cirrhosis related to either excessive alcohol consumption (80 g per day in males and 60 g per day in females for at least 10 years), chronic infection with HCV (serum HCV antibody-positive), HBV (serum HBsAg positive), and/or hereditary HFE1 haemochromatosis; and ii) the absence of previous hepatic complications. From June 2000 to March 2006, among the 1,340 randomized patients, 62 were subsequently excluded from analysis after revision of individual data due to either immediate loss to follow-up (n = 12) or to the presence of a focal liver lesion at inclusion (n = 50), leading to a total of 1,278 patients analysed. At least 1 focal lesion was detected in 358 patients (28%), but HCC was confirmed in only 123 (9.6%). US surveillance performed every 3 months detected more small focal lesions ≤10 mm than US every 6 months but did not improve the detection of small HCC. Patients were no longer followed for clinical research purposes after the publication of this study.

For the present study, we selected all participants with cirrhosis related to either excessive alcohol consumption (n = 517), chronic viral infection with HCV and/or HBV (n = 520) or both (n = 175) and excluded those with haemochromatosis. After a median follow-up of 57.5 months, 114 HCCs, 101 decompensations and 88 deaths without previous hepatic events were registered.

### ANRS CO12 CirVir

ANRS CO12 CirVir sponsored and funded by the ANRS (France Recherche Nord&Sud Sida HIV Hépatites) is a multicentre observational cohort that aims to characterize the incidence of complications of cirrhosis and to identify the associated risk factors using competing risks analysis.^13^ The full CirVir protocol is available via the ANRS Web site (http://anrs.fr).

Specific additional inclusion criteria were i) chronic infection with HCV and/or HBV regardless of the level of replication and alcohol consumption, ii) Child-Pugh A status, iii) absence of previous hepatic complications (particularly ascites, gastrointestinal haemorrhage, or HCC), and iv) absence of severe uncontrolled extrahepatic disease resulting in an estimated life expectancy of less than 1 year.

Patients were seen by physicians every 6 months, and the usual clinical and biological data were recorded. Missing biological data were determined on frozen serum samples provided by the CRB (Liver Disease Biobank, GroupeHospitalier Paris Seine-Saint-Denis BB-0033–00027). Doppler US examination was performed every 6 months. All events occurring during follow-up were recorded in a dedicated eCRF based on information obtained from patient medical files from each centre. Likely causes of death were established.

Among 1,822 patients recruited in 35 French clinical centres between March 2006 and July 2012, 1,671 were selected for further analysis. After an overall median follow-up of 69.2 months, 262 decompensations and 92 deaths without any previous hepatic event were registered. A definite diagnosis of HCC was established in 257 patients.

### CIRRAL

CIRRAL is a multicentre cohort study implemented in 22 French and 2 Belgian tertiary liver centres to capture the whole spectrum of complications occurring in compensated alcohol-related cirrhosis using competing risks analyses.^9^ The promoter was the APHP. The cohort was funded by the French National Institut of Cancer (INCa), the French Association for Research in Cancer and the ANRS (PAIR CHC 2009), and registered on ClinicalTrials.gov (NCT01213927). Specific additional inclusion criteria were i) cause of cirrhosis related to chronic alcohol abuse according to the World Health Organization criteria (more than 21 and 28 glasses per week for females and males, respectively) for at least 10 years, ii) absence of HBsAg or HCV antibodies, and iii) patients belonging to Child-Pugh A at enrolment. The follow-up of patients was strictly superimposed on the ANRSCO12 Cirvir cohort’s design.

Among 706 patients included between October 2010 and April 2016, 650 patients were selected for further analysis. After a median follow-up of 46.3 months, a definite diagnosis of HCC was established in 58 patients, and 105 decompensations and 68 deaths without previous hepatic events were registered.

### Statistical analyses

Analysis was performed at the reference date of November 18th, 2019, based on a database common to the 3 cohorts.

**Fig. 1:**
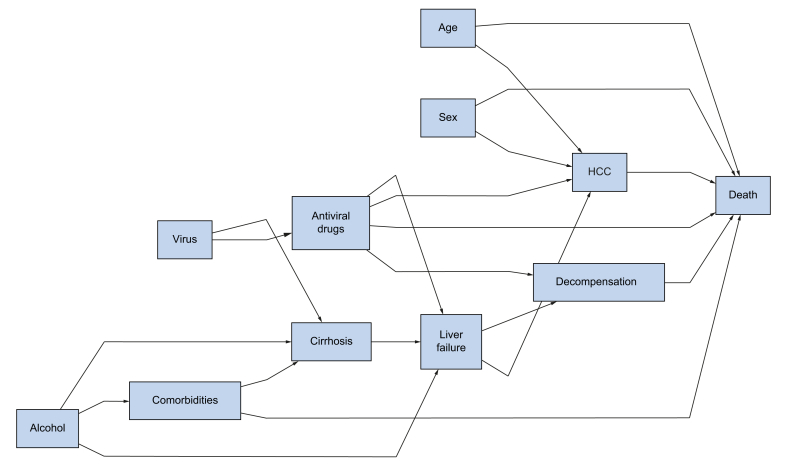
Direct acyclic graph. Both active viral replication and excessive alcohol consumption, alone or combined, may favour the development of cirrhosis. Several comorbidities (in particular, metabolic syndrome) further increase this risk and are more often associated with alcohol-related liver disease. Once cirrhosis is established, the progression towards liver failure may lead to both liver decompensation and HCC development. Such progression is dramatically decreased in HCV- or HBV-infected patients in whom sustained virosuppression can be achieved. Liver decompensation and subsequent end-stage liver disease, more frequently encountered in patients with alcohol-related cirrhosis, may act as competing risks of death, both before HCC development and following cancer management. HCC, hepatocellular carcinoma.

Summary statistics, *i.e.* absolute and relative frequencies or medians (IQRs) were computed. The cumulative incidence of HCC and decompensation (defined as the first occurrence of ascites, gastrointestinal haemorrhage, encephalopathy, or icterus before HCC) were estimated in a competing-risk setting, where death and liver transplantation free of the events of interest were considered to be competing events. Loss to follow-up was censored at the time of the last follow-up. Overall survival after HCC according to aetiology was illustrated by Kaplan-Meier curves. To deal with potential confounders from the literature and our expert knowledge of the disease, as illustrated by a direct acyclic graph (Fig. 1), Cox models and Fine and Gray models were used to compare outcomes (survival and cumulative incidences), adjusted for age and sex, across aetiological groups. Second, multivariate regression models were used to look for prognostic sets of variables, further including variables associated with the outcome at the 10% level from univariable analyses. Missing values of covariates were handled by multiple imputations with chained equations,^16^ based on M = 30 imputed complete datasets, with estimated hazard ratios (HRs) based on the average value of the regression coefficients.

Analyses were performed on SAS 9.3 (SAS Inc., Cary, NC) and R3.5.1 (http://www.R-project.org).

## Results

### Baseline characteristics of patients

A total of 3,533 patients (2,403 men, median age 55 years) were selected from the CHC 2000 trial (n = 1,212), ANRS CO12 CirVir (n = 1,671) and CIRRAL (n = 650) cohorts, pooled and split into 3 groups according to the aetiology of chronic liver disease (virus-related [VIR]: n = 1,926; alcohol [ALC]: n = 1,167; combined [MIX]: n = 440). Table 1 summarizes the baseline characteristics of the patients according to the aetiological groups. At enrolment, the cause of cirrhosis was controlled in a large portion of ALC patients (86% with daily alcohol consumption less than 2 glasses) but rarely in the VIR and MIX groups (21%), as only 272 out of 1,801 HCV patients had no baseline detectable serum HCV RNA (VIR 223, MIX 49), and 205 out of 474 HBV patients had no HBV DNA detectable (VIR 192, MIX 13).

**Table 1:** Baseline characteristics of the patients according to aetiology groups of the cirrhosis.

	n	Total (3,533 patients)	Viral (1,926 patients)	Alcoholic (1,167 patients)	Mixed (440 patients)	*p* value
Male gender	3,511	2,403 (68%)	1,238 (64%)	790 (69%)	375 (86%)	<10^−4^
Age, years	3,522	55 (48–64)	56 (48–65)	57 (50–64)	50 (45–57)	<10^−4^
BMI kg/ m^2^	3,014	26 (23–29)	27 (23–29)	27 (24–30.5)	25 (23–28)	<10^−4^
BMI ≧30 kg/m^2^	3,014	653 (22%)	303 (19%)	295 (29%)	55 (15%)	<10^−4^
Alcohol, g/day	3,187					<10^−4^
0–10		2,758 (86%)	1,608 (93%)	874 (81%)	276 (71%)	
11–50		259 (8%)	113 (7%)	84 (8%)	62 (16%)	
51–100		73 (4%)	0	73 (7%)	40 (10%)	
>100		45 (2%)	0	45 (4%)	12 (3%)	
Excessive alcohol consumption (years)	115	20 (19–29)	n.a.	20 (10–30)	10 (5–20)	<10^−4^
Smokers	2,716					<10^−4^
No		1,007 (37%)	758 (50%)	210 (24%)	39 (12%)	
Ex		694 (26%)	325 (22%)	291 (33%)	78 (24%)	
Current		1,015 (37%)	431 (28%)	375 (43%)	209 (64%)	
Diabetes	2,318	259 (11%)	93 (7%)	146 (23%)	20 (7.5%)	<10^−4^
History of liver decompensation	3,533	701 (20%)	30 (2%)	650 (56%)	21 (5%)	<10^−4^
Esophageal varices	2,899					<10^−4^
0		1,720 (59%)	1,047 (69%)	472 (45%)	201 (61%)	
1		742 (26%)	304 (20%)	350 (33%)	88 (27%)	
2		381 (13%)	146 (10%)	202 (19%)	33 (10%)	
3		56 (2%)	14 (1%)	37 (3%)	5 (2%)	
Prothrombin time %	3,339	84 (74–95)	88 (78–97)	76 (65–88)	86 (76–96)	<10^−4^
Albumin, g/ L	3,339	41 (38–44)	42 (39–45)	40 (36–43)	41 (38–45)	<10^−4^
Bilirubin, μmol/L	3,237	13 (9–18)	12 (8–16)	15 (10–24)	12 (8–18)	<10^−4^
ALT, IU/L	3,471	41 (25–77)	53 (31–96)	26 (19–39)	61 (34–102)	<10^−4^
AST, IU/L	3,461	43 (29–73)	49 (31–83)	35 (26–50)	54 (33–90)	<10^−4^
GGT, IU/L	3,418	82 (43–165)	67 (36–127)	106 (54–220)	102 (57–203)	<10^−4^
Platelets, Giga/L	3,416	138 (99–183)	140 (100–186)	139 (102–185)	125 (87–175)	0.0003
AFP, ng/ml	3,187	5 (3–8)	5 (3–10)	4 (3–6)	6 (3–10)	<10^−4^
MELD	2,536	8.3 (7.5–9.4)	7.9 (7.5–8.9)	8.9 (7.8–10.9)	7.9 (7.5–9.0)	<10^−4^
Child-Pugh A	3,255	3,128 (96%)	1,808 (99%)	924 (90%)	396 (98%)	<10^−4^
HIV +	3,078	78 (2.5%)	63 (3.4%)	2 (0.2%)	13 (3.4%)	<10^−4^
HCV antibodies	3,316					<10^−4^
Positive		1,801 (54%)	1,434 (78%)	0	367 (92%)	
Negative		1,515 (46%)	414 (22%)	1,069 (100%)	32 (8%)	
HCV RNA	2,540					
Positive		1,333 (52%)	1,059 (73%)	0	274 (72%)	0.85
Negative		1,207 (48%)	400 (27%)	701 (100%)	106 (28%)	1.00
HBsAg	3,406					<10^−4^
Positive		474 (14%)	437 (23%)	0	37 (9%)	
Negative		2,932 (86%)	1,446 (77%)	1,103 (100%)	383 (91%)	
HBV DNA	1,151					
Positive		173 (15%)	153 (35%)	0	20 (45%)	0.19
Negative		978 (85%)	280 (65%)	674 (100%)	24 (55%)	0.55
Fibroscan, kPa	1,364	16.5 (10.5–26.5)	14.5 (10–22)	24.5 (14–42)	17.5 (12–28)	<10^−4^

Categorical and binary variables are summarised by percentages-continuous variables are summarized using median (IQR). Univariate comparisons use Fisher's exact test for categorical variables and Wilcoxon rank test for quantitative variables. AFP, alpha-fetoprotein; ALT, alanine aminotransferase; AST, aspartate aminotransferase; GGT, gamma glutamyltransferase; MELD, model for end-stage liver disease.

### Outcome of patients during surveillance programs

At the reference date, the median (IQR) follow-up was 57.8 months (36–79.5), and differed in the VIR, ALC and MIX groups as follows: 62 (39–85), 40 (24–63) and 55 (31–77) months for the occurrence of decompensation; 62 (38–85), 42 (27–64) and 54 (30–77) months for the occurrence of HCC; and 65 (43–87), 43 (29–65) and 56 (34–79) months for overall survival.

**Fig. 2:**
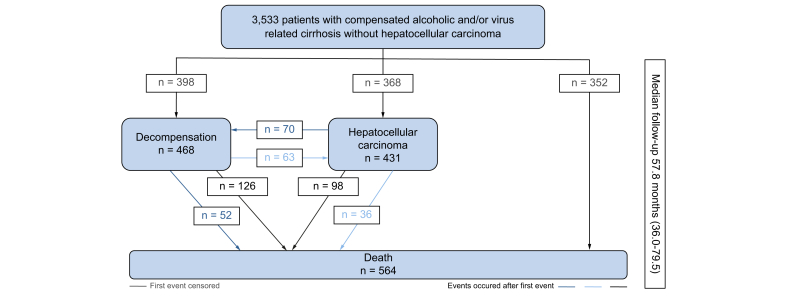
Outcomes in the whole population at the reference date of analysis (2019, November 18^th^).

**Fig. 3:**
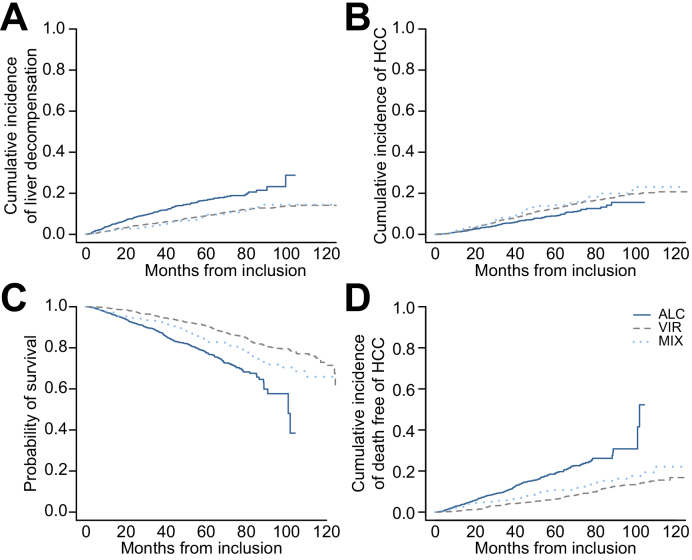
Outcomes of patients according to the aetiology of the liver disease. (A) The 1-year incidence of first decompensation and (C) the 5-year overall survival differed across groups, adjusting for age and sex (*p* <0.0001 for both comparisons, log-rank). (B) The cumulative incidence of HCC across aetiology groups also differed after adjusting for age and sex (*p* = 0.0011, log-rank), (D) together with different competing risks of death (*p* <0.0001, adjusted for age and sex, log-rank). HCC, hepatocellular carcinoma.

At the reference date, virological control was achieved in 1,100 patients (48%) (HCV RNA undetectable in 694 VIR and 146 MIX; HBV DNA undetectable in 242 VIR and 18 MIX), 431 patients had developed HCC (VIR 14%, ALC 8%, MIX 15%), 398 patients had experienced at least 1 episode of liver decompensation (VIR 189, ALC 169, MIX 40) and 564 had died (VIR 258, ALC 231, MIX 75) (Fig. 2). Fig. 3 displays the outcomes according to the aetiology of the underlying cirrhosis. The 1-year incidence of first decompensation (VIR 8.8%, 95% CI, 7.5–10.3; ALC 16.5%, 95% CI, 14.1–19.1; MIX 9.1%, 95% CI 6.3–12.6) and the 5-year overall survival (VIR 90.7%, 95% CI 89.3–92.1; ALC 77.6%, 95% CI 74.7–80.6; MIX 83.2%, 95% CI 79.2–87.4) differed across groups, after adjusting for age and sex (*p* <0.0001 for both comparisons), with causes of death mainly liver-related in VIR and MIX (HCC 53/258 [20.5%] and 10/75 [13.3%]; hepatic impairment 63/258 [24.4%] and 15/75 [20%]) and mainly related either to terminal liver failure (57/231 [24.7%]) or extrahepatic cancers (25/231 [10.8**%**] *vs.* 3/258 [1.2%]) in ALC. Similarly, the cumulative incidence of HCC across aetiology groups (5-year estimates: VIR 12.6%, 95% CI 11.0–14.2; ALC 8.9%, 95% CI 7.1–11.0; MIX 14.2%, 95% CI 10.8–18.1) also differed after adjusting for age and sex (*p* = 0.0011), together with different competing risks of death (5-year estimate of death before the occurrence of HCC: VIR 6.3%, 95% CI 5.2–7.6; ALC 19.1%, 95% CI 16.4–21.9; MIX 10.7%, 95% CI 7.7–14.2; *p* <0.0001, adjusted for age and sex). Table S1 and S2 report the patient characteristics associated with the cumulative incidence of liver decompensation and occurrence of HCC, respectively. The importance of viral aetiology in predicting decompensation was confirmed, while HCC occurrence was mostly related to age and sex.

### Characteristics of HCC diagnosed during systematic surveillance

The baseline characteristics of HCC are detailed according to the aetiological groups (Table 2, Table 3). At diagnosis, serum alpha-fetoprotein was significantly lower in the ALC (median, 5.4 ng/ml) than in the other groups (VIR 18, MIX 34 ng/ml; *p* = 0.0006). While tumour burden was comparable in terms of prevalence of single nodule (VIR 61%, ALC 55%, MIX 66%; *p* = 0.34), median diameter (VIR 20, ALC 21, MIX 18 mm; *p* = 0.92), macrovascular invasion (VIR 8%, ALC 13%, MIX 10%; *p* = 0.50), extrahepatic metastasis (VIR 4%, ALC 2%, MIX 8%; *p* = 0.55) and within Milan criteria (VIR 76%, ALC 68%, MIX 73%; *p* = 0.36), the prevalence of early BCLC stages (0/A) was significantly less frequent in patients with alcohol-related cirrhosis (VIR 80%, ALC 37%, MIX 72%; *p* <10^−4^) as their general condition (ECOG-performance status 0: VIR 89%, ALC 63%, MIX 85%; *p* <10^−4^) and liver function (Child-Pugh A: VIR 84%, ALC 74.6%, MIX 80%; *p* = 0.18) were more impaired.

**Table 2:** Main characteristics of HCC according to aetiology.

	n	Total (431 patients)	Viral (273 patients)	Alcoholic (95 patients)	Mixed (63 patients)	*p* value
**At HCC diagnosis**						
Male gender	429	309 (72%)	178 (65%)	79 (84%)	52 (83%)	0.0003
Age, years	430	63 (55–71)	64 (55–72)	65 (61–72)	55 (50–64)	<10^−4^
Median time between last normal liver imaging and HCC (months, IQR)		6.4 (3.3–10.2)	6.2 (3.4–9.1)	6.9 (2.9–12.6)	6.7 (3.8–11.0)	0.39
Solitary nodule	405	245 (61%)	158 (61%)	48 (55%)	39 (66%)	0.34
Size of main nodule, mm	377	20 (15–26)	20 (15–27)	21 (15–26)	18 (16–24)	0.96
AFP, ng/ml	211	10 (4–87)	18 (5.5–119)	5.4 (3.2–17.2)	34 (8–127)	0.0006
Macrovascular invasion	352	33 (9%)	18 (8%)	10 (13%)	5 (10%)	0.50
Extrahepatic metastasis	146	5 (3%)	2 (4%)	2 (2%)	1 (8%)	0.55
Performance status	329					<10^−4^
0		278 (84%)	203 (89%)	31 (63%)	44 (85 %)	
1–2–3		51 (16%)	25 (11%)	18 (37%)	8 (15%)	
Milan +	431	318 (74%)	207 (76%)	65 (68%)	46 (73%)	0.36
BCLC	317					<10^–4^
0/A		225 (71%)	166 (80%)	22 (37%)	37 (72%)	
B, C or D		92 (29%)	41 (20%)	37 (63%)	14 (28%)	
Prothrombin time, %	281					0.22
<40		7 (2%)	5 (3%)	2 (3%)	0	
40–50		9 (3%)	3 (2%)	5 (7%)	1 (3%)	
>50		265 (95%)	166 (95%)	64 (90%)	35 (97%)	
Albumin g/L	251					0.002
<28		18 (7%)	8 (5%)	5 (7%)	5 (14%)	
28–35		67 (27%)	28 (19%)	23 (35%)	16 (43%)	
>35		166 (66%)	112 (76%)	38 (58%)	16 (43%)	
Bilirubin μmol/L	250					0.50
<35		211 (84%)	122 (84%)	61 (87%)	28 (82%)	
35–50		25 (10%)	13 (9%)	7 (10%)	5 (15%)	
>50		14 (6%)	11 (7%)	2 (3%)	1 (3%)	
Child-Pugh B or C	360	66 (18%)	37 (16%)	18 (25%)	11 (20%)	0.18
Platelets G/L	244	111 (80–154)	111 (80–154)	125 (96–179)	111 (72–160)	0.47
Esophageal Varices	431					0.21
0		290 (67%)	192 (70%)	59 (62%)	39 (62%)	
>0		141 (33%)	81 (30%)	36 (38%)	24 (38%)	
**First-line treatment of HCC**						
Any treatment	431					0.14
Yes		393 (91%)	251 (92%)	82 (86%)	60 (95%)	
No		38 (9%)	22 (8%)	13 (14%)	3 (5%)	
Curative treatment*	431					0.57
None		188 (44%)	118 (43%)	45 (47%)	25 (40%)	
Percutaneous ablation**		186 (43%)	115 (42%)	42 (44%)	29 (46%)	
Resection		54 (12%)	39 (14%)	7 (7%)	8 (13%)	
Both		3 (1%)	1 (1%)	1 (2%)	1 (1%)	
Resection	417	57 (13%)	40 (15%)	8 (9%)	9 (15%)	0.34
Percutaneous ablation**	431	189 (44%)	116 (42%)	43 (46%)	30 (48%)	0.72
Arterial embolisation***	415	88 (21%)	54 (20%)	19 (22%)	15 (24%)	0.84
Sorafenib	414	42 (10%)	25 (9%)	12 (14%)	5 (8%)	0.40
Miscellaneous	290	37 (9%)	25 (9%)	2 (2%)	10 (16%)	0.035
Liver transplantation ***	301	32 (8%)	24 (9%)	2 (2%)	6 (10%)	0.094

Univariate comparisons use Fisher’s exact test for categorical variables and Wilcoxon rank test for quantitative variables. AFP, alpha-fetoprotein; BCLC, Barcleona Clinic Liver Cancer; HCC, hepatocellular carcinoma.

* Either resection or percutaneous ablation.

** radiofrequency, microwave, irreversible electroporation.

*** 2^nd^ intent after local treatment.

**Table 3:** Main characteristics of HCC diagnosed during periodical surveillance according to 2 aetiology groups of cirrhosis after pooling alcohol and mixed groups.

	n	Total (431 patients)	Viral (273 patients)	Alcoholic+Mixed (158 patients)	*p* value
**At HCC diagnosis**					
Sex (M/F)	429	309 (72%)	178 (65%)	131 (83%)	0.0001
Age (years)	430	63 (55–71)	64 (55–72)	63 (55–70)	0.29
Median time between last normal liver imaging and HCC (months, IQR)		6.4 (3.3–10.2)	6.2 (3.4–9.1)	6.9 (3.2–11.9)	0.18
Solitary nodule (n, %)	405	245 (61%)	158 (61%)	87 (60%)	0.76
Size of main nodule (mm)	377	20 (15–26)	20 (15–27)	20 (15–25)	0.87
AFP (ng/ml)	211	10 (4–87)	18 (5.5–119)	8 (3.7–57)	0.023
Macrovascular invasion	352	33 (9%)	18 (8%)	15 (11%)	0.42
Extrahepatic metastasis	146	5 (3%)	2 (4%)	3 (3%)	1.00
Performance status	329				0.001
0		278 (84%)	203 (89%)	75 (74%)	
1–2–3		51(16%)	25 (11%)	26 (26%)	
Milan +	431	318 (74%)	207 (76%)	111 (70%)	0.25
BCLC	317				<10^–4^
0/A		225 (71%)	166 (80%)	59 (54%)	
B,C,D		92 (29%)	41 (20%)	51 (46%)	
Prothrombin time (%)	281				0.18
<40		7 (2%)	5 (3%)	2 (2%)	
40–50		9 (3%)	3 (2%)	6 (6%)	
>50		264 (94%)	166 (95%)	99 (92%)	
Albumin (g/L)	252				0.0008
<28		18 (7%)	8 (5%)	10 (10%)	
28–35		67 (27%)	28 (19%)	39 (37%)	
>35		166 (66%)	112 (76%)	55 (53%)	
Bilirubin (μmol/L)	250				0.25
<35		211 (84%)	122 (84%)	89 (86%)	
35–50		25 (10%)	13 (9%)	12 (11%)	
>50		14 (6%)	11 (7%)	3 (3%)	
Child-Pugh B or C	360	66 (18%)	37 (16%)	29 (23%)	0.12
Platelets (G/mm^3^)	244	111 (80–154)	111 (80–154)	111 (79–170)	0.73
Esophageal varices (0–>0)	431	290/141	192 / 81	98/ 60	0.10
**First-line treatment of HCC**					
Any Treatment	431				0.58
Yes		393 (91%)	251 (92%)	142 (90%)	
No		38 (9%)	22 (8%)	16 (10%)	
Curative treatment*	431				0.36
None		188 (44%)	118 (43%)	70 (44%)	
Percutaneous ablation**		186 (43%)	115 (42%)	71 (45%)	
Resection		54 (12%)	39 (14%)	15 (10%)	
Both		3 (1%)	1 (1%)	2 (1%)	
Resection	417	57 (13%)	40 (15%)	17 (11%)	0.33
Percutaneous ablation**	431	189 (44%)	116 (42%)	73 (47%)	0.45
Arterial embolisation	415	88 (21%)	54 (20%)	33 (22%)	0.81
Sorafenib	414	42 (10%)	25 (9%)	17 (11%)	0.64
Miscellaneous	290	37 (9%)	25 (9%)	12 (12%)	1.00
Liver transplantation***	301	32 (8%)	24 (9%)	8 (5%)	0.23

Univariate comparisons use Fisher’s exact test for categorical variables and Wilcoxon rank test for quantitative variables. AFP, alpha-fetoprotein; BCLC, Barcleona Clinic Liver Cancer; HCC, hepatocellular carcinoma.

* Either resection or percutaneous ablation

** radiofrequency, microwave, irreversible electroporation

*** 2^nd^ intent after local treatment.

The median time between the last normal liver imaging and the diagnosis of HCC was 6.4 months (IQR 3.3–10.2) – without any significant difference according to aetiological groups (*p* = 0.39) – and did not impact on overall survival (*p* = 0.80). The distribution of the modalities of HCC screening did not significantly differ according to the aetiological groups.

The spectra of first-line HCC treatments and second-line transplantation are summarized according to the aetiological groups (Table 2, Table 3). Overall, 243 (56%) patients underwent first-line treatment with curative intent (resection 54, ablation 186, both 3). Despite more impaired general conditions and liver function in those with alcohol-related cirrhosis, the proportion of patients who received first-line curative treatment was not significantly different according to the aetiology of the underlying cirrhosis (resection: VIR 40 patients, ALC 8, MIX 9, ablation VIR 116 patients, ALC 43, MIX 30).

### Observed survival after HCC diagnosis and management

**Fig. 4:**
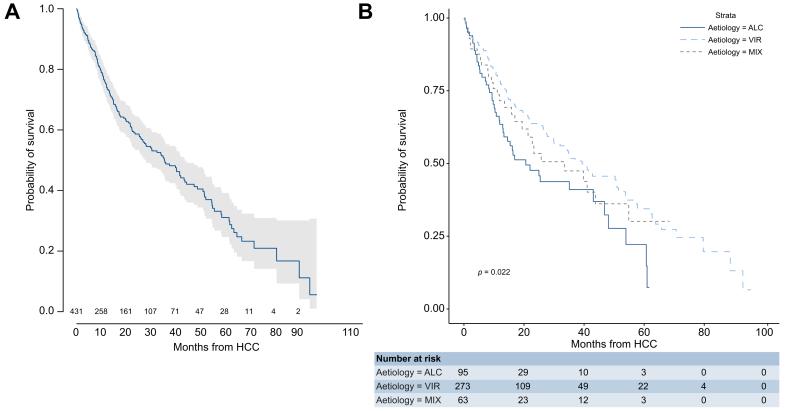
Overall survival after the occurrence of HCC. (A) Overall median survival (35 months; 95% CI 27.5–43.1) and (B) median survival according to the aetiology of cirrhosis (VIR 39, ALC 21, and MIX 34 months; *p* = 0.0045, log-rank). ALC, alcohol-related; HCC, hepatocellular carcinoma; MIX, alcohol and virus-related; VIR, virus-related.

Fig. 4 displays survival after HCC, with 186 (43%) observed deaths (VIR 111, ALC 47, MIX 28). The median survival was 35 months (95% CI 27.5–43.1) (VIR 39, ALC 21, MIX 34; 5-year estimates: VIR 34.4%, ALC 22.2%, MIX 30.1%). The hazard of death was decreased in the VIR group (HR 0.62; 95% CI 0.44–0.87) compared to the ALC group (Table 4). The main cause of death was decompensation in the ALC group but HCC progression in the VIR group.

**Table 4:** Univariable and multivariable Cox models for survival after HCC.

	n	Univariable			Multivariable		
		HR	95% CI	*p* value	HR	95% CI	*p* value
Gender							
Female	120	1.00					
Male	309	0.82	0.60–1.12	0.22			
Age, years	430	0.99	0.85–1.14	0.85			
BMI							
<30 kg/m^2^	274	1.00					
≧30 kg/m^2^	92	0.91	0.63–1.32	0.62			
Diabetes							
No	275	1.00					
Yes	41	1.28	0.78–2.1	0.33			
Smokers							
Ex	101	1.00					
Current	114	1.36	0.9–2.05	0.14			
No	131	1.30	0.88–1.94	0.19			
Comorbidities (obesity or diabetes or current smoker)							
None	194	1.00					
At least one	211	1.08	0.8–1.46	0.62			
Aetiology of cirrhosis							
Alcohol	95	1.00			1.00		
Virus	273	0.62	0.44–0.87	0.006	1.06	0.64–1.78	0.80
Mixed	63	0.74	0.66–1.19	0.21	1.00	0.53–1.91	0.98
Multi nodule	160	1.00					
Single nodule	245	0.52	0.39–0.71	<0.0001	0.94	0.63–1.41	0.77
Size of main nodule mm	**377**	**1.03**	**1.02–1.04**	**<0.0001**	**1.02**	**1.00**–**1.03**	**0.003**
AFP ng/ml	211	1.06	1.03–1.09	<0.0001	1.01	0.97–1.06	0.47
Performance status							
0	**278**	**1.00**					
1/ 2/ 3	**51**	**4.39**	**2.96–6.53**	**<0.0001**	**1.84**	**1.02**–**3.23**	**0.045**
Child-Pugh							
A	**294**	**1.00**					
B/C	**66**	**3.36**	**2.35–4.81**	**<0.0001**	**2.31**	**1.50**–**3.58**	**<0.0001**
BCLC							
0–A	**225**	**1.00**					
B–C–D	**92**	**5.71**	**3.98–8.17**	**<0.0001**	**2.17**	**1.05**–**4.47**	**0.04**
Platelets, G/L	244	1.00	0.99–1.0	0.25			
Esophageal varices							
Grade 0/1	290	1.00					
Grade 2/3	141	1.52	1.13–2.05	0.005	1.08	0.75–1.53	0.69

Univariate comparisons use Fisher's exact test for categorical variables and Wilcoxon rank test for quantitative variables. Independant prognostic factors are indicated in bold. AFP, alpha-fetoprotein; BCLC, Barcleona Clinic Liver Cancer; HCC, hepatocellular carcinoma; HR, hazard ratio.

### Effect of aetiology on survival following HCC detection

Among the prognostic variables identified by univariable analyses (Table 4), 4 independent factors were identified by the multivariable Cox model after imputation of missing values: size of the main tumour (HR 1.02, 95% CI 1.00–1.03, *p* = 0.003), performance status >0 (HR 1.84, 95% CI 1.02–3.32, *p* = 0.045), Child-Pugh score B or C (HR 2.31, 95% CI 1.50–3.58, *p* <0.0001) and BCLC stages B, C or D (HR 2.17, 95% CI 1.05–4.47, *p* = 0.04), while no significant impact of aetiology was observed.

## Discussion

This analysis of a large population of 3,533 patients with compensated cirrhosis, in whom 431 cases of HCC were prospectively diagnosed during the screening program, showed i) higher rates of non-HCC-related complications and death in patients with alcohol-related cirrhosis; ii) more frequently impaired general conditions (ECOG > 0) and/or liver function (Child-Pugh B or C) at the time of HCC detection in patients with alcoholic or mixed cirrhosis; iii) similar access to first-line HCC therapy with curative intent regardless of the aetiology of the underlying chronic liver disease; and iv) a median overall survival up to 35 months after the diagnosis of HCC, which differed according to the aetiology of cirrhosis.

As expected, HCC occurrence in patients with alcohol-related disease strongly competed with death compared to those with virus-related or combined cirrhosis. Such an observation is further reinforced by the growing rates of sustained virologic response and maintained virosuppression over the past few years in patients with virus-related disease, which have enabled a dramatic decrease in the incidence of non-HCC liver-related events in the last 10 years, as well as a potential reduction in extrahepatic mortality.^17^ This point is particularly illustrated by the long-term follow-up of patients from the CirVir cohort and CHC2000 trial, which covered several antiviral therapeutic eras between 2000 and 2016. Nevertheless, these high rates of competing deaths should not rule out HCC screening in patients with alcohol-related cirrhosis. In addition to controlling the cause of liver disease, patients with alcoholism are less likely to undergo surveillance than those with virus-related cirrhosis because of the large proportion with undiagnosed cirrhosis and/or the lower uptake of HCC surveillance in patients with diagnosed cirrhosis, leading to late diagnosis of HCC.^18^^,^^19^ These delays are thought to result in a larger tumour burden at diagnosis and poorer outcomes in patients with alcohol-related liver disease than in those infected by HCV.[19], [20], [21] Nevertheless, our analyses based on longitudinal follow-up suggest that compliance with surveillance was similar in all patients regardless of the aetiology of cirrhosis, as assessed by the time elapsed with the last normal liver imaging techniques before HCC diagnosis. This point constitutes a positive message to encourage physicians to implement surveillance programs in patients with alcohol-related cirrhosis.

Moreover, our results suggest the benefits of HCC screening in patients who were primarily diagnosed at early HCC stages. Indeed, when diagnosed in the context of a strict surveillance programme, HCC prognosis appears mostly dictated by liver function and general conditions regardless of the aetiology of the underlying cirrhosis. Opposite to ITA.LI., in the CA cohort,^20^ we did not find that HCC tumour burden at diagnosis was influenced by aetiology. The latter indeed only considered patients at the time of HCC diagnosis, which may explain, at least in part, such a discrepancy. This fact highlights the importance of longitudinal assessment of surveillance programs to support confidence in the conclusions drawn.

A roughly similar access to curative therapy regardless of the aetiology of the underlying cirrhosis (57% as a whole) has to be noticed. Percutaneous ablation was the most frequent curative procedure applied in all patients, highlighting the utility of these techniques, particularly in patients with comorbidities or compromised liver function and/or portal hypertension. Nevertheless, despite similar access to curative procedures, patients with alcohol-related HCC had shorter survival in the long term, which could illustrate the complex interplay between lower access to sequential HCC treatment due to progression of liver failure, as well as non-liver-related causes of death.

The main strengths of this study are i) the homogeneity of the population as all patients had biopsy-proven and baseline compensated cirrhosis; ii) the large sample size of patients with compensated cirrhosis who developed HCC during the surveillance program; and iii) the prospective follow-up according to a standardized surveillance schedule. Conversely, our study has several limitations. Some confounding factors in the comparison of outcomes are likely. Thus, we used multivariate regression analysis to limit confounding by aetiological bias. We retrospectively combined data from 1 randomized controlled trial and 2 cohorts, mostly to increase the external validity of our findings; however, this may have influenced the results by introducing some heterogeneity in patient populations, procedures, other patient management and data quality. In addition, we did not include the use of antiviral therapies in this analysis given that the main purpose of the paper was to compare patient outcomes according to the aetiology of cirrhosis and given that their use is meaningless in alcoholic patients.

In conclusion, HCC surveillance seems to be effective in patients with alcohol-related cirrhosis, leading to similar access to curative therapy as for those with viral cirrhosis. Nevertheless, higher rates of comorbidities and faster progression of hepatic failure strongly influence the outcome. In the case of HCC detected during follow-up, these 2 parameters also impact long-term survival, irrespective of the initial tumour burden and aetiology of cirrhosis.

## Financial support

The promoters of the prospective 3 cohorts were the Assistance Publique des Hôpitaux de Paris (APHP) for CHC200 and CIRRAL and ANRS for CirVir. The cohorts were funded by i) CHC 2000: the French Ministry of Health (PHRC 1998 and 2003) and the French Ligue de Recherche contre le Cancer , ii) the 10.13039/501100003323National Agency for Research on HIV and Hepatitis (ANRS) for CirVir-iii) CIRRAL: the 10.13039/501100006364French National Institut of Cancer (INCa), the French 10.13039/100007391Association pour la Recherche sur le Cancer and the ANRS (PAIR CHC 2009).

## Authors’ contributions

*Study concept and design:* Nathalie Ganne-Carrié, Pierre Nahon, Sylvie Chevret. *Acquisition of data :* Nathalie Ganne-Carrié, Pierre Nahon, Cendrine Chaffaut, Gisèle Nkontchou, R. Layese, E. Audureau, Sylvie Chevret. *Analysis and interpretation of data:* Cendrine Chaffaut, Sylvie Chevret, Nathalie Ganne-Carrié, Pierre Nahon. *Drafting of the manuscript:* Nathalie Ganne-Carrié, Pierre Nahon, Sylvie Chevret. *Critical revision of the manuscript for important intellectual content:* Cendrine Chaffaut, Sylvie Chevret, Nathalie Ganne-Carrié, Pierre Nahon, E. Audureau, G. Nkontchou. *Statistical analysis:* Cendrine Chaffaut and Sylvie Chevret. *Administrative, technical and material* support*:* Cendrine Chaffaut, Sylvie Chevret. *Study supervision:* Cendrine Chaffaut, Sylvie Chevret, Nathalie Ganne-Carrié, Pierre Nahon, E. Audureau.

## Data availability statement

Data available on request to SBIM, Hôpital Saint-Louis, Paris, (sylvie.chevret@u-paris.fr).

## Conflict of interest

Prof. Ganne-Carrié received honoraria from Bayer, Gilead, Ipsen and Shionogi. Prof Nahon has received honoraria/grants from 10.13039/100006483Abbvie, 10.13039/100004325AstraZeneca, 10.13039/100004326Bayer, 10.13039/100002491Bristol-Myers Squibb, 10.13039/100005564Gilead and 10.13039/501100014382Ipsen.

Please refer to the accompanying ICMJE disclosure forms for further details.
