# Supplementary material for: Impact of cirrhosis aetiology on incidence and prognosis of hepatocellular carcinoma diagnosed during surveillance
Source: JHEP Rep. 2021 Mar 26;3(3):100285. doi: 10.1016/j.jhepr.2021.100285 (PMC8424277; doi:10.1016/j.jhepr.2021.100285)
Supplement: Multimedia component 1 [file mmc1.pdf]

Impact of cirrhosis aetiology on incidence and prognosis of hepatocellular carcinoma diagnosed during surveillance

Nathalie Ganne-Carrié, Pierre Nahon, Cendrine Chaffaut, Gisèle N'Kontchou, Richard Layese, Etienne Audureau, Sylvie Chevret for CIRRAL and CirVir groups

Table of contents

Table S1..2

Table S2..3

Table S1. Univariable and multivariable Fine and Gray models for decompensation

	n	Univariable			Multivariable		
		HR	95%CI	p	HR	IC 95%	p
**Gender**							
- **Female**	1108	1.00			1.00		
- **Male**	2403	0.79	0.64-0.97	0.022	0.79	0.62-1.01	0.058
**Age**, years	3522	1.01	1.00-1.02	0.0047	1.00	0.99-1.02	0.48
**BMI**							
**< 30 kg/m^2**	2361	1.00					
**≧ 30 kg/m^2**	653	1.00	0.77-1.30	0.99			
**Diabetes**							
**No**	2059	1.00			1.00		
**yes**	259	1.58	1.15-2.16	0.0043	1.37	0.96-1.96	0.084
**Smokers**							
**Ex**	694	1.00					
**Current**	1015	1.12	0.84-1.48	0.44			
**No**	1007	0.94	0.71-1.25	0.66			
**Comorbidities** (obesity or diabetes or current smoker)							
**None**	1642	1.00			1.00		
**At least one**	1662	1.24	1.01-1.52	0.039	0.98	0.75-1.28	0.87
**Etiology of cirrhosis**							
- **Alcohol**	1167	1.00			1.00		
- **Virus**	1926	0.53	0.43-0.65	<0.0001	0.61	0.46-0.80	0.0003
- **Mixed**	440	0.50	0.36-0.72	0.0001	0.74	0.49-1.10	0.14

95%CI: 95% confidence interval

Table S2. Univariable and multivariable Fine and Gray models for HCC

	n	Univariable			Multivariable		
		HR	95%CI	p	HR	IC 95%	p
**Gender**							
- **Female**	1108	1.00			1.00		
- **Male**	2403	1.26	1.02-1.55	0.033	1.56	1.25-1.94	<0.0001
**Age**, years	3522	1.03	1.02-1.04	<0.0001	1.04	1.03-1.05	<0.0001
**BMI**							
**< 30 kg/m^2**	2361	1.00					
**≧ 30 kg/m^2**	653	1.25	0.99-1.59	0.06			
**Diabetes**							
**No**	2059	1.00					
**yes**	259	1.24	0.89-1.72	0.20			
**Smokers**							
**Ex**	694	1.00					
**Current**	1015	0.83	0.64-1.09	0.18			
**No**	1007	0.81	0.62-1.05	0.11			
**Comorbidities** (obesity or diabetes or current smoker)							
**None**	1642	1.00					
**At least one**	1662	1.15	0.95-1.40	0.16			
**Etiology of cirrhosis**							
- **Alcohol**	1167	1.00			1.00		
- **Virus**	1926	1.40	1.10-1.76	0.005	1.43	1.13-1.81	0.0028
- **Mixed**	440	1.56	1.13-2.14	0.006	1.80	1.30-2.48	0.0004

95%CI: 95% confidence interval-
